# The Radiological Assessment of Root Features and Periodontal Structures in Endodontically Treated Teeth Subjected to Forces Generated by Fixed Orthodontic Appliances. A Prospective, Clinical Cohort Study

**DOI:** 10.3390/jcm10102078

**Published:** 2021-05-12

**Authors:** Katarzyna Pustułka, Agata Trzcionka, Arkadiusz Dziedzic, Dariusz Skaba, Marta Tanasiewicz

**Affiliations:** 1 Department of Conservative Dentistry with Endodontics, Faculty of Medical Sciences in Zabrze, Medical University of Silesia, Plac Akademicki 17, 41-902 Bytom, Poland; swistekk@wp.pl (K.P.); atrzcionka@sum.edu.pl (A.T.); martatanasiewicz@sum.edu.pl (M.T.); 2 Department of Periodontology, Faculty of Medical Sciences in Zabrze, Medical University of Silesia, Plac Traugutta 1, 41-800 Zabrze, Poland; dskaba@sum.edu.pl

**Keywords:** orthodontic treatment, endodontics, fixed orthodontic appliances, external root resorption, periodontal structures, periodontal space, radiological assessment

## Abstract

The various side effects of orthodontic treatment with fixed orthodontic appliances (FOAs) and their impact on apical and periodontal structures have been widely reported. However, the existing data is not yet conclusive. Aims and objectives: To investigate the status of roots and periodontium in endodontically treated teeth that have undergone orthodontic treatment with the use of FOAs and to evaluate their impact on apical/periodontal structures. Material and methods: The prospective clinical cohort study initially involved 69 participants aged 16–40, without underlying systemic conditions, who received orthodontic treatment with ligatureless FOA systems due to different types of mild and moderate malocclusions. To meet the required criteria, 88 teeth in 34 patients were assessed clinically and radiologically. Participants had at least one tooth treated endodontically while the corresponding tooth from the same anatomical group on the opposite side was vital and intact (a ‘split-mouth’ approach). Four cohorts were allocated: Group IA consisted of 15 teeth, treated utilising the principles of modern endodontics, that were subjected to orthodontic forces no less than six months after completing the root canal obturation. Group I consisted of 13 similarly endodontically treated teeth, which commenced orthodontic treatment at least six months after the completed endodontic therapy. Group II contained 16 teeth treated by conventional endodontic methods and the control group, Group III, contained 44 clinically and radiologically intact teeth (incisors and premolars) with vital and sound dental pulp. The response of apical and periodontal structures to FOAs was determined by data collected from intraoral periapical radiographs taken within the course of five consecutive appointments during the orthodontic treatment. Results: No statistically significant differences were observed in susceptibility to FOA-induced external apical root resorption (EARR) between combined Groups IA + IB and II. An association was, however, demonstrated, between the occurrence of EARR and the degree of expansion of the periodontal ligament (PDL) space, regardless the method of root canal treatment. Cumulative data revealed a positive correlation between the width of the PDL space and the stage of FOA treatment (the third and the fourth appointment). The subtle changes in radiological length of roots have been observed (min 0 mm/max 0.38 mm), particularly between the second and third appointment in Group II (*p* < 0.05). Conclusions: The standard orthodontic therapy with FOAs is a safe option with predictable outcome for persons who have recently received endodontic therapy. The anterior teeth, predominantly incisors, were more susceptible to minimal EARR than premolars, which suggests that the rate of EARR occurrence may depend upon the original morphology of the apical portion of the root. The use of additional orthodontic forces increases the risk of EARR and is associated with a higher incidence of radiologically detected PDL space widening.

## 1. Introduction

The potential side-effects of modern orthodontic treatment and its unfavourable impact on dentition and hard tissues have been extensively discussed in professional literature for decades. This scientific interest equally concerns teeth with normal dental pulp condition and those that have been subjected to endodontic treatment procedures. 

It has been postulated that teeth following the completion of endodontic treatment (non-vital teeth) exhibit a similar/same micromovement rate as vital ones that possess an intact dental pulp [1,2,3]. However, it is still disputable whether non-vital teeth are more prone to pathological disturbances of their apical root structures, including periodontium [1,2,4]. Wickwire et al. [5] claim that endodontically treated teeth (ETT) are more prone to external apical root resorption (EARR) and similar results were obtained by Spurrier et al. [2]. However, these differences were not detectable on a purely clinical level [1] and other studies carried out by Young et al. described no significant difference in the prevalence of apical complications after orthodontic treatment between sound and ETT [6]. Interestingly, Bender et al. [3,7] observed more cases of EARR in vital teeth compared with those treated endodontically. 

A review of existing literature demonstrates an evolution in the information relating to ETT being subject to orthodontic therapy with fixed orthodontic appliances (FOAs). Early data stated that this cohort is generally more prone to periodontium alterations and clinicians claimed that an endodontically treated tooth may behave as a foreign body, continuously irritating adjacent surrounding tissues, causing and initiating its EARR. In addition, and supported by other authors, it was also speculated that resorption usually results in ankylosis which prevents successful orthodontic treatment [7,8]; however, the results of studies published later (after 1980) did not show that relationship [3,7]. Recent publications have described the opposite effect, where teeth treated using modern endodontic methods rarely underwent EARR [6,8,9,10,11,12,13]. The clinical cohort studies, which involved teeth with periapical lesions that were treated simultaneously with endodontic and orthodontic methods demonstrated that orthodontic treatment only insignificantly diminishes the healing ability of those periapical lesions and does not have a negative effect on the roots and condition of the periodontal structures [14].

In prospective observational studies conducted in the early 1990s, the authors observed that even though intact and vital teeth were more prone to root resorption, the loss of length (average 0.77 mm) did not affect the integrity and clinical quality of the tooth [2]. These results concluded that equally intact, vital and non-vital teeth following endodontic therapy can be successfully treated with FOAs without major complications; similar observations were reconfirmed by Remington et al. [2,15]. Additionally, Spurriere et al. showed that there was no association between patient’s gender and the severity of apical malformations in endodontically treated teeth; however, such dependence was observed in vital teeth which seemed more prone to resorption in males [2]. Interestingly, Moh et al. stated that there was no difference in tooth movement between vital and non-vital teeth as using an animal model they observed a higher number of resorptive lacunae on the lateral surfaces of ETT [7,16]. It has been suggested that these lesions might be linked to a loss of vitality and root canal treatment rather than orthodontic forces. Those observations were partially coherent with the early research of Mattison et al. (1984) [1,6].

Currently, as can be determined by the limited available evidence, it is assumed that teeth treated endodontically when utilising the latest endodontic methods and properly disinfected, 3D obturated and coronally restored teeth are less prone to EARR [9]. This is likely also related to coronal integrity, a lack of coronal leakage and an inhibited bacteria penetration, which may cause local inflammation leading to EARR. Hypothetically, the calcium hydroxide used during the course of root canal treatment may also prevent the initiation of EARR and periodontium damage [14]. This interim intracanal material with odontotropic ability is capable of alkalising the local tissues. Increased pH levels and the neutralisation of the acids that appear during the inflammation process that accompanies orthodontic movements of teeth activates alkaline phosphatase contributing to alveolar bone regeneration [14,17,18]. There is, however, a higher risk of resorption if the root is characterised with atypical morphology [7].

Hamilton and Gutaman claimed that traumatised teeth requiring endodontic treatment may be successfully moved with minimal risk of EARR, unless the resorption began before the commencement of orthodontic therapy, in which case the orthodontic forces may intensify the resorption process [7]. If the root canal treatment was performed because of inflammation or necrosis caused by caries, there are no contraindications to orthodontic treatment, provided that the periapical tissues are healthy. If signs of chronic periapical inflammation are present on the radiographs, orthodontic treatment should be postponed until the periapical condition improves as long as the time that has elapsed since the beginning of the root canal treatment is less than six months [19]. After that point the forces used during the course of orthodontic treatment may be the same as those generated by FOAs applied in vital teeth, e.g., 50–100 g for parallel movement of the mesial incisor [2,19].

The hypotheses regarding the negative impact of orthodontic treatment on the condition of ETT have evolved. Currently, it is assumed that endodontic treatment decreases the risk of EARR [2,7,8,9,10,11,12,14,19] and that properly selected and applied orthodontic forces do not negatively influence the apical and periodontal structures after root canal treatment, as long as that treatment was conducted according to current standards of endodontics, based on European Society of Endodontology (ESE) recommendations. It has been established that the condition of dental pulp plays an important role in the aetiology of root resorption and/or the remodeling of the root surface. In non-vital teeth, less activity of the odontoclast cells responsible for resorptive damage was observed [20].

Relatively scant and non-homogeneous scientific information has been produced concerning clinical investigations assessing the impact of orthodontic therapy with fixed appliances on periapical and periodontal structures in ETT when different endodontic methods were used. As there is limited availability of longitudinal and interventional clinical studies, it is uncertain whether patients with recently completed endodontic treatment can commence orthodontic treatment due to reported side effects affecting apical and periodontal tissue. As the expected benefits of orthodontic treatment should outweigh the potential risk of adverse events, the consent for a proposed treatment must include the likelihood of side effects, as some of them are believed to be related to ETT. The lack of uniform and explicit interdisciplinary guidelines often prevents the timely orthodontic therapy in situations when root canal treatment is required due to pulp pathologies. Therefore, there is an urgent need to establish transparent recommendations supporting orthodontists to provide treatment with the use of FOAs in light of timing rationale and clinical safety of endodontic therapy.

The aim of this study was to provide a comprehensive, multi-level radiological evaluation of root features and periodontium changes in endodontically treated teeth when they are exposed to orthodontic treatment (OT) with the use of ligatureless FOAs. The primary rationale of the research was to assess the impact of FOA treatment on apical and periodontal structures and draw conclusions regarding the safety, as well as the rationality of orthodontic treatment with FOAs in patients who had completed endodontic treatment using both conventional and advanced methods. The obtained results will underpin the modern guidelines in both modern orthodontics and endodontics, providing support for clinicians and reassurance for patients.

## 2. Materials and Methods

### 2.1. Cohort Selection and Sample Distribution

The prospective, ‘split-mouth’ approach, clinical cohort study was conducted on 69 participants aged 16–40 who underwent orthodontic treatment with conventional FOAs (private dental practice StomArt) between 2013 and 2015. Individuals were either in a growing stage or had finished their skeletal growth, confirmed by analysis of cervical vertebrae profiles from lateral teleradiographs of the cranium [21]. The orthodontic treatment was carried out following the general clinical principles of orthodontic correction of malocclusion in adults. It consisted of the standard orthodontic camouflage of skeletal malocclusions or the correction of appendicular teeth defects. Each patient possessed at least one endodontically treated tooth exposed to FOA forces. The patient, intervention, comparison and outcome questions were established during an initial phase to provide clear goals of the study, justifying clinical intervention and the research protocol:

*p*-adolescents and adults aged 16–40.

Intervention orthodontic treatment with conventional fixed orthodontic appliances.

Comparison endodontically treated teeth exposed to FOA forces were compared to intact, sound teeth on opposite site in the same arch.

Outcome the impact of FOA-induced forces on roots, apical and periodontal structures, primarily incidents of external root resorption and PDL space changes.

**Figure 1:**
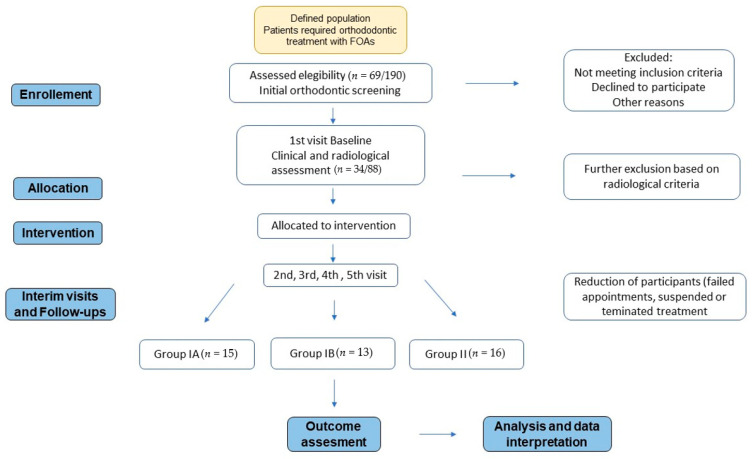
Structured protocol of prospective cohort study. Flow diagram.

The clinical protocol of the study was approved by the ethics committee of the Medical University of Silesia (KNW/0022/KB/121/10). The study was conducted according to the guidelines of the Declaration of Helsinki and fully adhered to the Strengthening the Reporting of Observational Studies in Epidemiology (STROBE) protocol for methodological quality assessment of observational studies [22], in accordance with Enhancing the Quality and Transparency of Health Research (EQUATOR) [23]. These standardised reporting checklists are intended to improve the comprehensiveness and transparency of research project associated with clinical cohort studies. The STROBE checklist is attached as supplementary material (Table S1). Structured and rigour study protocol is presented on Figure 1.

The clinicians, experienced registered specialists in endodontics and orthodontics, were involved in the development and design of the study in relation to outcome measure, feasibility, acceptability and the recruitment of study samples. The study methods were critically appraised during the drafting of the protocol, following optimisation to adhere to current clinical and research guidelines. All data were handled in accordance with up-to-date legislation, including the Data Protection Act 2018. Physical and digital data were anonymised and stored to only be accessible to the research team. After participants’ follow-up, all data were stored for five years.

Valid informed written consent was obtained from the participants or their legal guardians (for patients were 18 years old). Allocation of the patients to particular groups and the assessment of the oral cavity status were determined on the basis of radiological criteria, medical history, intraoral examination. Whilst the approach of this interdisciplinary cohort study is novel there is scanty comparable data available for a sample size calculation. The sample estimation was performed by using the difference between independent means to calculate the number required to reject the null hypothesis that the parameters means of cohort and control groups are equal with specific probability. As the data loss was unavoidable, when collecting follow-up data, the sample number was adjusted for the estimated drop-out rate. The targeted and allocated groups of participants are described in Table 1

The assessment of endodontically treated teeth status was conducted during the orthodontic review appointments. The standardised clinical and radiological assessment protocol is presented in Table 2. The random allocation of ETT to the groups ‘conventional’ and ‘modern’ endodontics methods (root canal preparation and obturation) was applied. The conventional endodontic methods involved: Endodontic hand files, step-back preparation technique, standard irrigation with antimicrobial agents and single gutta-percha point or multiple gutta-percha points ‘cold’ lateral condensation for root canal obturation, with the use of root canal sealers. The modern endodontic methods involved: Rotary Ni-Ti files, crown-down technique, standard irrigation with antimicrobial agents and thermoplastic root canal obturation methods (‘heated’), with the use of root canal sealers.

### 2.2. Eligibility Criteria

#### 2.2.1. Inclusion Criteria

- Patients aged 16–40 treated with FOAs and had at least one endodontically treated tooth
- Good general systemic health, no underlying medical conditions, not on medications for chronic diseases or affecting bone/connective tissue structures
- No active periodontal diseases
- No removable prosthodontic appliances
- Optimal oral hygiene
- Valid informed written consent obtained as a compulsory requirement for participation in the study

#### 2.2.2. Exclusion Criteria

- Patients with underlying chronic medical conditions, either acquired or congenital
- Pregnancy
- Patients with allergy to any dental material used
- Multiple missing posterior teeth
- Periodontal diseases
- Temporomandibular disorder and bruxism
- Oral mucosal diseases
- Persons who were not dentally fit before the orthodontic treatment
- Patients who have already been treated with the FOAs
- Poor oral hygiene
- Medications that can affect the efficiency and outcome of the orthodontic treatment: Bisphosphonates, steroids, calcium supplementation
- Unable to select a control, intact tooth
- Periapical index score above 1

#### 2.2.3. Minimising Chance Error, Bias and Confounding

**Figure 2:**
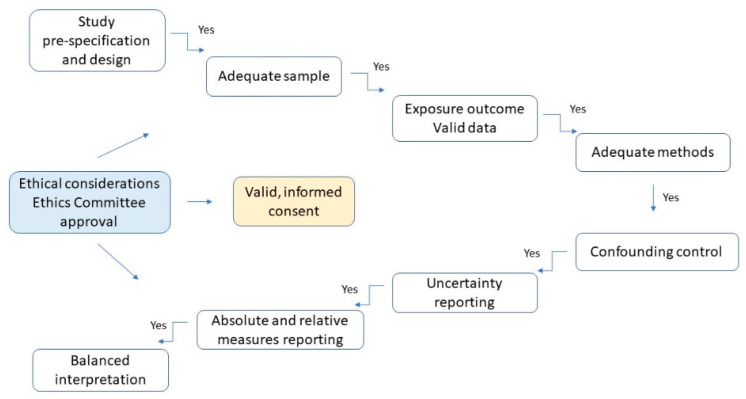
The verification process of prospective study credibility and protocol. Applied flowchart.

The potential study bias and error were minimised by ensuring a representative sample, reliable, standardised outcome measuring and using the adequate statistical data analysis. Group demographics was relatively homogenous, with equal distribution of independent determinants. The final version of clinical and research protocol was verified by main supervisor, a consultant in conservative dentistry with endodontics, registered specialist following an agreement with a registered specialist in orthodontics. The summary of study verification and protocol approval process is presented on Figure 2.

### 2.3. Orthodontic Treatment Protocol and Clinical Stages

All patients were managed by a single practitioner, a registered specialist in orthodontics. Treatment was conducted by fitting FOAs onto both upper and lower arches using the straight arch technique. Selected patients were treated with the low-friction system. The following two different types of ligatures braces were used:

- 022 MC Carriere MBT (metal)
- 022 Clarity S.L. MBT (cosmetic)

The subsequent clinical stages of the orthodontic treatment were as follows:

1st Alignment 014 or 016 NiTi Kinetix, then 016 × 025 NiTi Kinetix

2nd Active phase 017 × 025 POSTED or BetaCNA as working archwire

3rd Finishing and stabilisation 019 × 025 Multiforce as finishing archwire

The additional orthodontic appliances were used in selected cases, if clinically justified by fitting mainly intraoral inter-arch tractions, class II and III or less commonly Hyrax/Hass fixed appliances.

The follow-up orthodontic appointments took place on regular basis, every few months. The treatment course was completed with fixed retention phase in lower arch. A metal retainer wire attached was for three years onto the lingual surfaces of the lower anterior teeth (canine–canine). In the maxillary arch retention was provided for two years using a removable retainer made from a clear thermoplastic material.

### 2.4. Radiographic Examination

The radiological assessment protocol was approved by the ethics committee as a crucial element of the study protocol. This stage is consistent with the ESE recommendation for a follow-up period of up to five years after completion of endodontic treatment. A repeated periapical intraoral radiograph, preferably digital, is recommended as a postoperative means of monitoring the long-term outcome of endodontic therapy. This is particularly important during orthodontic treatment with FOAs. The main operator and clinical supervisor of the study were both calibrated prior to radiological data collection. The clinical supervisor of the study was a consultant in conservative dentistry and endodontics.

The clinical follow-up of endodontically treated teeth started with an intraoral periapical radiograph. A series of five subsequent radiographs was a primary measure for radiological assessment of apical portion of the root and periapical structures, i.e., periodontal ligament (PDL) space width [24]. During the follow-up visits, accordingly the first, third and fifth, extraoral orthopantomogram radiographs (OPTs) were taken [9,25] in order to verify the teeth alignment, braces positioning and the periapical status of the treated teeth [9].

To thoroughly assess the periodontal structures’ status, the standardised intraoral periapical radiographs (IPRs) were taken using the parallel technique which facilitated replication and reproductivity of the image with the same exposing parameters. In accordance with the study protocol, the essential dental radiographs were taken using modern, digital dental radiography equipment: A Kodak 2200 intraoral X-ray machine, equipped with the RVG 6100 Kodak digital system and Kodak 800 orthopantomography extraoral digital scanner.

The precise and replicable positioning was executed using KERR Super-Bite Sendo film holders to obtain the same exposure conditions and eliminate the potential errors related to distortions and/or disproportional alterations, e.g., elongation or shortening of the tooth structures [26,27]. A standard protocol for ionising radiation protection in dental practice was strictly followed. All stages of the clinical protocol related to radiological assessment were approved by the ethics committee as an essential part of the project. The radiological evaluation was initially calibrated for two clinicians who subsequently validated the data obtained from radiological findings.

### 2.5. Analysis of the Intraoral Periapical Radiographs of Endodontically Treated Teeth

The IPRs that fulfilled the following criteria were assessed:

- The whole tooth structure was visible, from the root apex to the incisal/occlusal edge;
- the alveolar bone was fully projected, along with apical and marginal periodontal structures [2,28].
- The specific parameters of apical and periodontal elements taken into consideration during a radiological assessment of ETT were:
- apical range and homogeneity of root canal obturation (only for initial IPRs assessment to select/exclude cases);
- root’s radiological length (mm);
- periodontal space width (PAI scoring system, Ostravik criteria);
- shape and morphological profile of the root (Mirabella and Artun criteria);
- presence/absence of apical external root resorption (Levander and Malmgren criteria).

#### 2.5.1. Apical Range and Homogeneity of Root Canal Obturation

**Figure 3:**
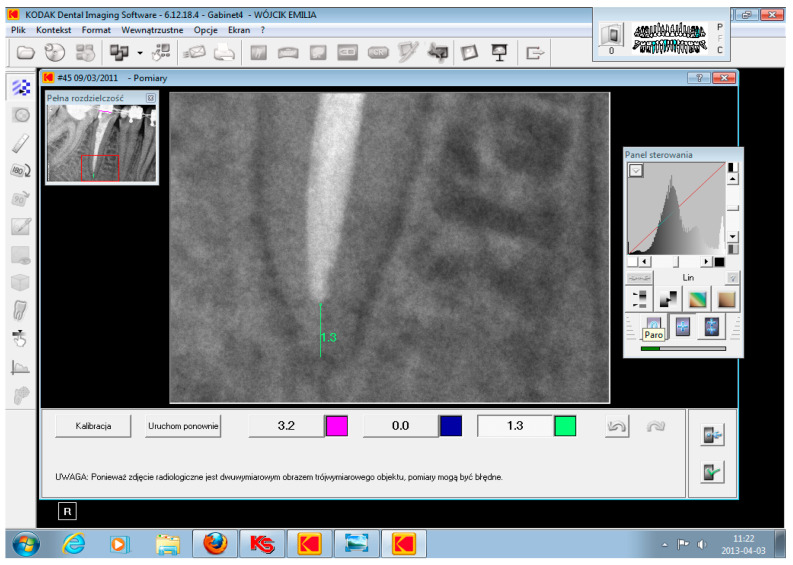
The screenshot of intraoral periapical radiograph (root’s apex magnification) of endodontically treated tooth presenting the measurement of the root canal filling range.

The initial calibration of radiographic software dedicated for IPRs was conducted using digital zoom and linear measurement options. The range of the canal filling on the initial IPR was measured using dedicated, digital Kodak imaging software. The distance between the radiological root apex and the termination of the root canal filling (radiopaque material) was measured (mm units). This initial procedure enabled the assignment of patients to one of the examined groups. The accuracy of the root canal filling range was assessed using micro-scale grading 0.1 or 2 (Figure 3). The second factor that influences the quality of the root canal filling is homogeneity of root canal obturation, defined as the uniformity of the filling material structure in the whole volume of the root canal. This parameter was assessed using a point scale of 0–2.

#### 2.5.2. Roots Radiological Length

**Figure 4:**
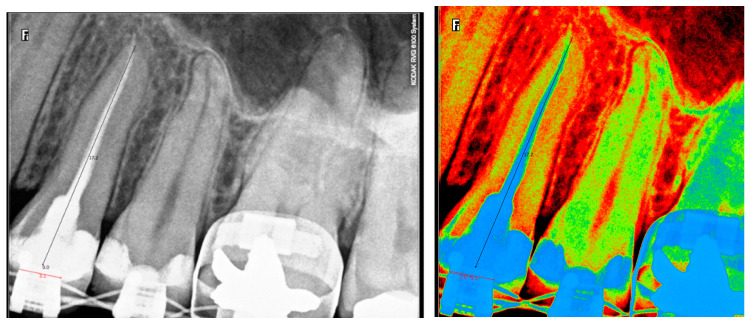
Digital measurement of radiological length using IPRs.

The standardised IPRs taken during each follow-up visit facilitated replication and reproductivity of the image with the same exposing parameters. The radiological length (mm) was measured using digital Kodak software dedicated for dental radiographs, following initial calibration. The measurement involved two reproducible landmarks: A radiological root’s apex end point and the margin of orthodontic bracket. This second landmark has been chosen instead of incisal edge or coronal cusp due to the fact that they might be affected by traumatic injuries, in the form of enamel chipping or incisal edge/cusp fracture or attrition (Figure 4). This parameter was calibrated by two operators.

#### 2.5.3. Periapical Structures and Periodontal Ligament Space Assessment Periapical Index

**Figure 5:**
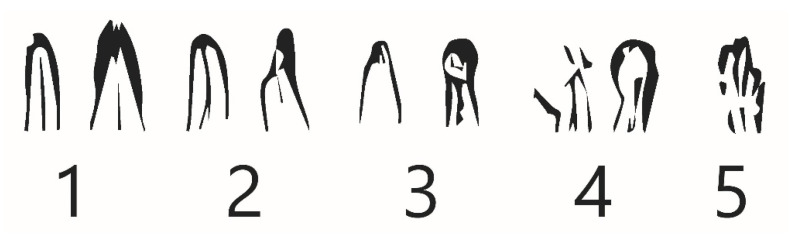
Periapical Index grading 1–5 scale (Ostravik 5-point scale).

To assess radiologically the response of periapical tissues to forces generated by FOAs and the status of periapical structures, including periodontal ligament space, the periapical index (PAI) grading was used (5-point ordinal scale, Ostravik, Figure 5), determined by radiological evaluation of reference intraoral periapical radiographs [29]:

PAI grade 1 normal periapical structures/features (healthy)

PAI grade 2 small changes in bone structure

PAI grade 3 structural bone changes with some mineral loss

PAI grade 4 periodontitis with a well defined radiolucency

PAI grade 5 apical radiolucency with bone expansion

The PAI scoring system is commonly used in retrospective studies of treatment results in endodontics, in clinical trials and epidemiological studies. When assessing the radiological changes in periapical area and PDL space during the second, third, fourth and fifth appointment, the initial IPRs were analysed because of the high discrepancy in width of PDL space, as well as the fact that PDL space may differ considerably from individual to individuals [30]. Both resorptive and regenerative processes caused by the orthodontic treatment commence in the periodontium. The monitoring of apical and periodontal structures by analysis of periapical radiographs provides crucial information regarding the changes occurring in periapical tissues during orthodontic treatment with FOA’s.

#### 2.5.4. Assessment of the Root Morphology

Due to the evident link between root’s morphology, anatomical structure and predisposition to EARR [9,19,24,30,31,32,33,34], evaluation of the root’s shape and its anatomical variations was conducted. Baselineradiological assessment of root apex was carried out using standard IPRs taken during the first appointment and employing the *Mirabella* and *Artun* criteria. A standardised comparison of root apex morphological features was conducted during the subsequent appointments. Changes in the root apex shape/profile indicated pathological and/or adaptational alterations in the form of EARR

#### 2.5.5. External Apical Root Resorption Assessment Levander and Malmgren Scale

**Figure 6:**
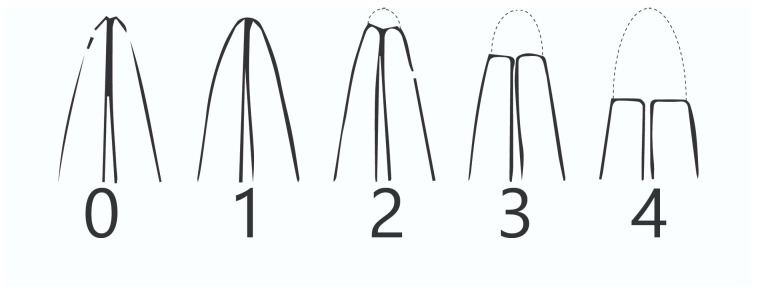
External apical root resorption classification (Levander and Malmgren grading 0–4).

In the presented study, the external apical root resorption was assessed using periapical radiographs. The OPGs only supported the baseline screening performed prior to main assessment. The scale developed by Levander and Malmgren was used during radiological assessment of IPRs [24,33,34,35]. The following grading criteria were applied (Figure 6):

1. No changes in root shape or surface (no resorption, intact external outline)
2. Irregular root contour/outline, root length has not yet been altered
3. Minor root resorption apically, limited to maximum of 2 mm (<2 mm)
4. Severe resorption from 2 mm to one-third, ¼ of the root length has been resorbed
5. Extreme resorption, exceeding one-third of the original root length.

In most cases, the EARR can be diagnosed accidentally when analyzing the OPGs. In order to obtain the general information on the periapical/periodontal structures and roots’ morphology, it is recommended to take panoramic radiographs before and after the orthodontic treatment course.

### 2.6. Statistical Analysis

Quantitative data distribution analysis was performed using the non-parametric Shapiro-Wilk test. To test the significance of differences between five dependent samples, ANOVA analysis and the Wilks’ lambda statistical tests were applied. To compare the independent samples, the ANOVA method, along with the NIR test was used. Missing data were not removed from analysis. Distribution of tooth length measurements was determined by a considerable asymmetry; hence, non-parametric tests were utilised.

## 3. Results

### 3.1. Participants and Material Characteristics

After the initial selection of 69 patients, 190 teeth were included for further assessment and investigation. Subsequently, following detailed radiological clinical and assessments during follow-up visits, 88 teeth in 36 participants were selected and met strict required inclusion criteria. They were divided into three study groups IA, IB, II (Table 1 and Table 2) and a control Group III. Subgroups IA and IB were also combined into a single group, Group IA + IB, described as ‘teeth treated using modern endodontics methods (Table 3). After the first, ‘baseline’ appointment following an initial assessment, a fraction of the original participants did not meet the strict inclusion criteria after a thorough clinical and radiological evaluation. The sample size decrease was caused by unavoidable drop-outs due to various reasons, such as: Failed to attend, orthodontic treatment termination, changes in oral or general health status. Overall, 36 of the 88 examined teeth were premolars and the rest incisors (52).

### 3.2. The Impact of Orthodontic Treatment Duration

The analysis of the IPRs in the IA and IB groups proved that there was no statistically significant difference in the occurrence of EARR, changes in radiological length of the tooth and changes in PDL space width throughout the course of orthodontic therapy. It was established that time that had elapsed from the completion of the endodontic treatment to the commencement of the FOA therapy did not affect the tooth’s response to the orthodontic forces.

### 3.3. Changes in Radiological Length of ETT

Based on the measurements of the radiological length of the ETT taken at the fifth appointment, the changes to this parameter were calculated and recorded. In all examined groups only a minimal shortening of the radiological length was observed, considered as a margin of error. However, the changes in radiological length of teeth in the IA + IB group were more noticeable over subsequent follow-up visits. In Group II the only differences between the second and the third examinations were statistically significant, in contrast to values between the third and fourth, and the fourth and fifth examinations (Table 4).

### 3.4. Changes of PDL Space Width in ETT

**Figure 7:**
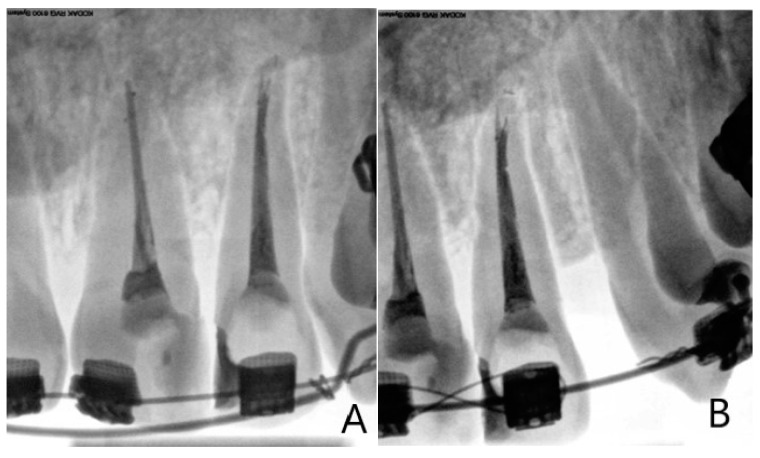
Intraoral periapical radiographs demonstrating the changes in periodontal ligament space width (Kodak software negative/positive mode to enhance subtle periodontal space changes. (**A**) Baseline intraoral periapical radiograph taken at the first appointment. (**B**) Intraoral periapical radiograph taken at the third appointment during treatment with FOAs. Evident widening of periodontal ligament space of distal/lateral aspect.

The subtle changes in PDL space width in 44 ETT obtained during the five control appointments are presented in Table 5. In order to assess the PDL space width, the standardised PAI index was used. The protocol included teeth without pathological changes in periapical tissues on the first appointment when the FOAs were fitted (Figure 7).

During the course of orthodontic treatment, the maximal noted PAI score was 3 (structural bone changes with some mineral loss). Results proved that in both examined groups the PAI values were higher the longer the orthodontic treatment lasted and the more intense the orthodontic forces. In the last stage of the orthodontic therapy (stabilisation), between the fourth and fifth appointments, the PAI values decreased and they returned to score PAI = 1 in selected cases. In the third and fourth measurements, the distribution of PAI values was similar in both examined groups. Only in seven radiographs (at the third appointment) was the PAI assessed as 3 (five from the IA + IB group; two from Group II). The most frequently obtained PAI score was 2, observed in 25 (57%) of ETT-54% from the IA + IB group and 62% from Group II (fifth appointment). At the last appointment, a PAI score of 1 was recorded in 50% of the IA + IB group cases and in 56% of Group II cases. The rest of the examined teeth scored a PAI of 2.

During the radiological examination at the second appointment, 61% of cases the IA + IB group had a PAI score of 2 or 3, with a corresponding occurrence in only 37% of teeth in Group II (Table 6). In Group II the changes in PDL space appeared gradually and slower in the first stage of the orthodontic treatment. From the third appointment the reaction of periodontal structures to the orthodontic forces was not apparent and detectable.

### 3.5. Morphological Profile of Root Apex

Out of the 44 ETT, 12 presented with an atypical original root shape (radiological assessment) in the form of flat root apex (7) and curved apex (5).

### 3.6. The Assessment of Root Apex Resorption Using the Lavender and Malmgren Scale

Table 7 and Table 8 present the data regarding root apex resorption in 44 ETT, on which the orthodontic forces generated by the FOAs were applied. In order to be included in the study, the Levander and Malmgren scale requires a tooth to score zero during the initial appointment (FOA fitting).

The cumulative number of EARR incidents in ETT occurred in 10 of the 44 examined teeth (22.7%). The first radiological signs of EARR were observed early usually after four months (second appointment): Four cases (9%), three in the IA + IB group and one in Group II. During the third appointment (after 8–10 months) there were six more cases (three in each examined group) totaling 10 cases. This number remained constant to the end of the study (Table 7).

**Figure 8:**
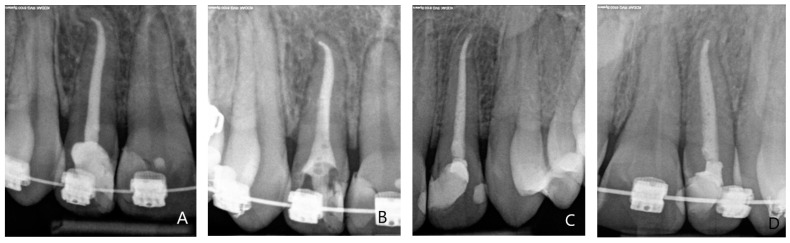
An anterior intraoral periapical radiograph taken during treatment with fixed orthodontic appliances. (**A**,**C**) Baseline intraoral periapical radiograph taken at the first appointment: Endodontically treated lateral maxillary incisor with atypical root shape (root apex curvature), maxillary central incisor (vital), with a typical morphology of the root. (**B**,**D**) Intraoral periapical radiographs taken at the third/fourth appointment: Mild external apical root resorption of lateral maxillary incisor and flattening of the apex of central maxillary incisor.

The consolidated results of the EARR assessment in relation to the duration of FOA treatment, the root apex morphology, anatomical group and the use of additional orthodontic forces are presented in Table 8. Detailed analysis of the data revealed that 50% of investigated teeth with EARR occurrence (5 of 10) had an atypical root apex morphological shape, mostly flat or curved. Twelve of 44 teeth had an atypical root shape. EARR was noted in only one premolar, with the remainder (90% of cases; nine of ten cases) occurring in incisors. In all the incisors included in the study, the presence of EARR was detected in 34.6% (nine of 26), while only 5.6% (one of 18) of posterior teeth developed various EARR, usually mild degree (Figure 8A–D). Overall, 80% of EARR cases were subject to additional orthodontic forces.

The statistical analysis (Table 9) revealed that the differences in EARR occurrence between the IA + IB group and Group II were not statistically significant. Hence, the susceptibility of those teeth to EARR was similar.

### 3.7. The Effect of the Application of Additional Orthodontic Forces Applied

Combining the results of this study, which showed that 80% of teeth with EARR were exposed to additional orthodontic forces, with the fact that higher susceptibility to EARR is usually observed in teeth loaded with higher orthodontic forces, two new groups were selected: A ‘YES’ group (with additional forces) and a ‘NO’ group (without additional forces) (Table 10).

All cases with EARR were allocated into a single group where the method of root canal treatment was not considered due to low number of observed cases. The statistical analysis clearly showed that teeth with additional orthodontic forces were diagnosed more frequently with EARR (Table 10). The results obtained during the second appointment had close tendency to statistical significance (*p* = 0.076) and after the third examination that difference was statistically significant (*p* = 0.032).

Results obtained during the second and third appointments (Table 11) showed that the prevalence of PDL space variation is attributed to the anatomical group of the tooth. Moreover, additional orthodontic forces are linked to a higher rate of PDL space enlargement detected on the radiographs. During the second appointment, an enlargement of the PDL space was noted in 70% of teeth in the ‘YES’ group, whilst similar changes were observed in 37.5% of teeth allocated to ‘NO’ group (*p* < 0.05). Following radiological examinations, an increase in the number of teeth PAI score of 2 or 3 was noted, with PAI scores of 3 occurring only in ‘YES’ cases (highly statistically significant). In the ‘NO group’, cases with PAI score of 3 were first observed during the fourth appointment (statistically significant difference). It can be concluded that the application of clinically justified additional orthodontic forces influence the dynamic rate of PDL space enlargement and PDL space width discrepancy in general. In contrast, the PDL not exposed to additional orthodontic forces seemed to enlarge with a slower rate and to a lesser degree and returned more rapidly to its initial state.

## 4. Discussion

Predictable orthodontic treatment should involve the application of near-to-physiological forces that are able to promote effective teeth movement (rotation, torque, tilting) with minimal risk to the root, periodontal structures or surrounding alveolar bone. This primary goal can be achieved by continuously applying low-grade force throughout the course of treatment [35,36]. This biologically favourable therapeutic mode is well tolerated by the natural periodontal structures, particularly periodontal cells responsible for PDL remodeling [36]. Interestingly, if the adequate orthodontic forces are applied continuously without major intervals, the damage to the PDL microvessels and subsequent tissue hypoxia can be avoided [37,38]. When conventional forces are used, the microvessels constriction takes place between each control appointment. This is a consequence of arch wire activation, sudden pressure towards the alveolus socket and localised compression of the periodontium, including the blood vessels. Recovery of the blood supply, which includes allowing the PDL space to be re-vascularised, may take a significant amount of time [36]. In fact, if the time for PDL remodeling and regeneration is not properly maintained in the course of the orthodontic treatment, the desired outcome of the treatment will be prolonged. This is particularly significant in adult patients where regeneration processes of periodontal structures are already markedly delayed [38].

In order to verify the hypotheses that composed the primary idea of the study, the detailed evaluation of radiographs taken during each of the follow-up appointment was carried out. We aimed to observe discrepancies in radiographs that could influence tooth functionality [39,40]. It was determined that orthodontic treatment with FOAs, even those generating low, near physiological forces, may cause foci of resorption of the root cementum at the compressed site [41,42]. These lesions can be observed in early stages of orthodontic treatment and up to one month after the orthodontic treatment commences [43], as micro-pathologies continue to accumulate throughout the treatment period [44].

While initial resorption lacunes visible in microtomography (micro-CT) cannot be diagnosed using standard dental radiographs, they do not influence tooth functionality and integrity [42,45]. In the literature they are referred as ‘natural scars’, which occur occurred following orthodontic treatment with FOAs [40]. Interestingly, these types of microscopic foci of resorption are also observed on the surfaces of teeth that have never been exposed to orthodontic treatment. This is considered an example of dynamic processes that take place in vital tissues of the human body regardless of their origin and location [33]. According to Matison et al., greater prevalence of microlacunes observed on lateral surfaces of the endodontically treated teeth is directly connected with loss of vitality, rather than with the orthodontic treatment itself [1,25]. This finding seems coherent with the results of other studies [1,6,46]. 

Moreover, an in-depth assessment of 220 dental radiographs revealed no effect of FOA treatment that was begun after the completion of endodontic treatment on a periodontal response to FOAs. Similar results were obtained by Leach et al., who claimed that there were no contraindications for the immediate start of orthodontic treatment following root canal therapy provided due to pulp or apical tissue inflammation as long as the periapical tissues appeared healthy [9,19].

In this study, only a minimal reduction of the root’s length (min 0.1–0.4 max median) was observed while comparing radiographs findings during orthodontic treatment. The predominant shortening of the root was observed during the second appointment in Group IA + IB (3.6 mm). In the group of teeth treated with conventional techniques, the maximal noted value was 2.2 mm and it was recorded during the fourth appointment. Similar results, showing very slight shortening of the root (0.77 mm) was observed by Spurrare et al. in their research on 43 patients. They concluded that observed discrepancy did not influence the tooth integrity [2] and presented results that were also confirmed by Renington et al. [15]. Therefore, a tooth may become increasingly clinically mobile if the reduction of its root exceeds 9 mm [19,32].

Smale et al. and Lavender and Malmgren stated that EARR during FOA treatment was relatively frequent, although in most cases only a slight alteration in the root contour without its shortening was observed [9,31,32]. Comparatively, EARR signs were detected in 10 out of 44 of the ETT overall (*p* > 0.05, no statistical significance compared to control). Susceptibility to EARR study groups was the same; hence, our thesis that teeth treated with the newest endodontic methods are less susceptible to EARR [9]. It must be highlighted that all teeth included in our study were asymptomatic when the FOA was fitted and scored 4–6 out of six points following root canal obturation quality assessment.

When assessing the PDL space width in endodontically treated teeth, we observed that regardless of the endodontic treatment, the changes to the PDL space were reasonably predictable and characteristic [47]. The intensification of orthodontic forces subsequently increased PAI score. In the last stage of the orthodontic treatment (stabilisation), the PAI values decreased; however, no case repeated its score of 1. In both groups, the PAI scores were a maximum of 3 out of 5; however, in Group II the enlargement of the PDL space dynamic was slower. A noticeable difference in the acceleration of PDL space enlargement was observed in the first 4–6 months, which may explain the fact of later EARR occurrences in Group II. During the second appointment with Group IA + IB, there were three cases of EARR and PAI ≥2 was observed in 61% of teeth. In Group II, there was only one case of EARR, with PAI ≥2 observed in 38% of teeth. Our own results support the evidence that EARR usually occurs in the early stage of orthodontic treatment [45,46]. Moreover, the statistical analysis confirmed a positive correlation between EARR occurrences and PDL space with increase. This relationship has also been confirmed by other researchers [41,42].

It is vitally important for the orthodontist to detect any case of apical EARR observed during active stages of the orthodontic treatment. This situation may indicate an initial sign for the requirement to start the passive (lasting 3–6 months) stage of treatment, with periodical radiological assessment of the teeth with suspected EARR [19,48]. Superficial changes to the root structure can completely regenerate when the orthodontic forces are removed [42,49]. Winter et al. claimed that the treatment’s interval duration can enhance the regenerative process [50]. The mild enlargement of PDL space observed during FOA treatment is usually manageable as the PAI scores lower than 3 assumed to be physiological when they occur during the orthodontic treatment and reversible when the treatment terminates. According to the available data, the anterior teeth, primarily the incisors, are the most susceptible to apical root resorption [51], contrast to posterior mandibular ones [19,31]. This can be explained by multidirectional movements in all three planes during orthodontic treatment. For the upper lateral incisors, an additional factor increasing susceptibility to resorption was deemed occurrence of deviations in the root shapes [33].

Here, present study showed higher susceptibility to EARR of teeth belonging to Group I, with these fractional results: Anterior 34.5% vs posterior 5.5%. These results are in agreement with other studies which observed root resorption in 25% of incisors during the course of the orthodontic treatment [52]. However, it has to be noted that only in 2.3% of cases was the root length discrepancy above 4 mm. It can thus be concluded that, hypothetically, if the root resorption is not present in the upper incisor, the likelihood of its occurrence in other anatomical groups of teeth is very low [53]. In addition, it was demonstrated that the orthodontic forces applied to the endodontically treated incisors were linked frequently with PDL space widening, as observed on dental radiographs, compared to premolars. Moreover, slightly increased mobility of both endodontically treated and non-treated teeth during the orthodontic treatment was observed. It has been previously elucidated that atypical root shape/morphology is a contributing factor to EARR in all teeth, regardless of the group they belong to [31,32,33].

The causes of root structure damage based on the type and power of forces used in orthodontic treatment were also analyses [41,44]. Beck and Harris confirmed that if low force was applied, the root apex resorptions were less extensive [54]. Other studies concentrated on the pattern of the orthodontic forces; specifically, whether there was constant or intermittent. It was concluded that the intermittent forces induced by the additional elements were attributed to the root resorption occurrence [31,45]. In addition, the type of malocclusion did not have an obvious impact on the occurrence of roots apex resorption. However, it was noticed that hypofunctional teeth were more prone to root resorption; this could be explained by less apparent PDL space and its lower vascularisation in general.

Essentially, the age of patients exposed to orthodontic treatment with FOAs is deemed a potential risk factor of intraoperative and postoperative complications. Due to the higher bone density and lower vascularity in adults, it is more challenging to activate the cellular elements, namely osteoblasts and osteoclasts and alter periodontal structures in a favourable direction. As a result, those physiological determinants are responsible for unstable and impaired tooth micro-movements in adults [55]. Therefore, more intense forces must be applied, along with prolonged treatment times. Unfortunately, however, this can increase the risk of adverse effects occurring [45]. Harris and Becker claimed that this problem was specific to adults with other predisposing factors such as chronic peripheral inflammation or atrophic changes in periodontal tissues [55]. The latest studies showed that the risk of localised periodontal tissue damages can be diminished by providing FOA treatment in stages, with appropriate breaks between the control dosing of the orthodontic forces without reaching their critical values [41,42]. The association between the type of slot, technique modality/intensity and EARR susceptibility was not observed. Similarly, Mohandesan et al. found no significant correlation between EARR and treatment technique, although it was correlated with gender for the lateral incisors [56].

Study revealed that additional orthodontic forces applied could be attributed to higher susceptibility to EARR compared with conventional orthodontic arch wire use. Those results were statistically significant (*p* = 0.032) and are consistent with the existing evidence that the presence of additional orthodontic forces has a positive correlation with PDL space enlargement.

Summarising, the results of the study can warrant the feasibility of early orthodontic intervention with FOAs considering the non-contributory impact of endodontic treatment on long-term clinical outcomes. Patients who need orthodontic treatment for various reasons require a clear assertion that it is safe to continue with FOA therapy even if they had recently completed root canal treatment.

### Limitations

The strict protocol’s criteria for observational studies were set up to avoid specific confounding factors that may hinder the results on different levels and with varying magnitude. In this project, non-significant variables were collected at baseline and an assessment for confounders was carried out. The randomisation, sampling, homogeneous groups and clear inclusion/exclusion criteria are deemed the main positive significant predictors of the high methodological quality of observational study. 

The outcome and risk of FOA-induced side effects towards teeth strongly depends on individual determinants, such as PDL susceptibility to damage/regeneration is linked to genetics, bone remodeling and tissue predisposition. The applied force generated by FOAs may not be comparable and reproducible and its use relies solely on orthodontist’s decision.

The sample size, i.e., number of participants was matched with the minimum required criteria for prospective, interventional, clinical studies. From a purely statistical point of view, the sample size selection of a clinical cohort study depends on the type of intervention and realistic approach when collecting clinical data. Considering practical aspects of long-term orthodontic courses with FOAs, usually extended for a minimum of 2–3 years, the collection of required data can pose a real challenge to research teams. Whilst the approach of this interdisciplinary cohort study is novel there is scanty comparable data available for a sample size calculation. The complex clinical cohort studies can be prone to suboptimal number of participants due to the nature of treatment course and obvious limitations related to prolonged therapeutic protocols. Despite this, the obtained results were thoroughly verified and reflect representative samples.

Additional radiological evaluation, including a 3D Cone Beam Computed Tomography (CBCT) assessment could also support the methodological reliability and data validation. Due to radiation protection regulations, this modern diagnostic imaging possesses obvious limitations, particularly when applied in young age group. It needs to be stressed that the focal resolution of the CBCT method may not be sufficient for detailed examination and measurement of subtle changes within apical/periodontal tissue. Unquestionably, the intraoral periapical digital dental radiographs currently remain the ‘gold standard’ for in-depth radiological assessment of apical/periodontal structures. However, their interpretation can differ, depending on the operator’s experience, image enhancement mode and settings.

Nevertheless, the results from well designed limited-size interventional studies vastly enrich the existing information about the clinical safety of orthodontic interventions. Further randomised control studies, with double-blind verification of clinical, radiological findings and large-size cohorts would enhance the data’s validation. An appropriate sample size will provide reproducibility and underpin the validity of future observational clinical research in interdisciplinary dental studies.

## 5. Conclusions

Orthodontic treatment with FOAs in adolescents and middle-aged individuals has a mild, reversible impact on periodontal and apical root structures of teeth when subject to conventional orthodontic forces. The predisposition occurs most commonly in single-rooted anterior teeth. Orthodontic treatment with FOAs when commenced immediately after the completion of endodontic treatment, does not increase the risk of external root resorption. Susceptibility to EARR as a result of FOA use is not determined by endodontic treatment techniques. Hence, the orthodontic therapy with fixed orthodontic appliances is deemed as a safe option in patients who have completed endodontic therapy soon before malocclusion correction.

Conventional orthodontic treatment with FOAs increases the potential risk of PDL and apical periodontium alterations, regardless of the endodontics technique utilised. The PDL space changes occur throughout the orthodontic treatment’s course due to compensating mechanisms. However, they do not indicate a predilection to the initiation of external root resorption. The additional orthodontic means and forces may exacerbate the susceptibility to external root resorption affecting apical and periodontal structures.

## Figures and Tables

**Table 1:** Characteristics of participants’ groups.

Group		Description and Details
I	A	Asymptomatic teeth without periapical pathologies, endodontically treated according to ESE guidelines Radiologically scored using the ESE criteria (4–6 points) Orthodontic treatment started 0–6 months after the endodontic treatment was completed
	B	Asymptomatic teeth with no periapical pathologies, endodontically treated according to the modern ESE guidelines Radiologically scored using ESE criteria 4–6 points. Orthodontic treatment started 6 months after the endodontic treatment was completed
IA + IB		Asymptomatic teeth without periapical lesions, endodontically treated using modern endodontics Radiologically scored using ESE endodontic criteria: 4–6 points
II		Asymptomatic teeth without periapical pathologies, treated with conventional endodontic methods
III		Asymptomatic, control, intact teeth at the same patients as the one fter endodontic treatment, Corresponding teeth, same anatomical group on the opposite site in the same dental arch

**Table 2:** Clinical stages of orthodontic protocol and assessment.

	Stage of the Study	Clinical Stage	Description
1	I	Orthodontic consultation	Dental and medical history Extra and intraoral examination Functional examination Analysis of the facial features (photo) Dental impressions for the diagnostic models
2		Orthodontic treatment plan	Analysis of diagnostic models and photos Analysis of extraoral radiographs: Orthopantomogram and teleradiography Orthodontic treatment plan set up
3		Selection of patients	Assessment of intraoral periapical radiographs of endodontically treated teeth Assessment of oral health status and systemic health of the patients included Assessment of control tooth status
4	II	Endodontic treatment	Performed following ESE standards
5	III	Orthodontic treatment	Fitting of the fixed orthodontic appliance Regular follow-ups and recalls
6	IV	Regular review of teeth selected for the study	Clinical and radiological assessment performed during five review appointments
7		Verification of the investigated group	Assessment of the quality of intraoral periapical radiographs Elimination of teeth not fulfilling the criteria
8	V	Data analysis, results validation, conclusions	Statistical analysis of the obtained data Discussion of the results, Conclusions

**Table 3:** Characteristic of study material in allocated groups.

Group	Initial Number of Patients	Initial Number of Teeth	Final Number of Patients	Final Number of Teeth
*IA*	69	95	34	15
*IB*				13
*II*				16
*III*		95		44

**Table 4:** Changes in radiological length of endodontically treated teeth (mm) at subsequent appointments.

Visit		Group IA + IB Median (Min–Max) {Quartile Lower Upper}	Group II Median (Min–Max) {Quartile Lower Upper}	Mann-Whitney Test (*p*)
**2nd**		0.2 (0−3.8) {0.05−0.4}	0.1 (0−1.2) {0−0.35}	*0.4642*
**3rd**		0.3 (0−3.6) {0.05−0.8}	0.35 (0−1.2) {0.05−0.7}	*0.9611*
**4th**		0.3 (0−3.5) {0−1.2}	0.3 (0−2.2) {0.1−0.9}	*0.7053*
**5thANOVA** **Friedman (*p*)**		0.2 (0−3.5) {0−1.3} 0.1771	0.4 (0−2) {0.1−1.2} 0.0276 *	*0.5664*
**Comparison between next visits Wilcoxon test**	**2nd** **3rd**	_____	P = 0.0446 *	
	**3rd** **4th**	*_____*	*P = 0.4326*	
	**4th** **5th**	*_____*	*P = 0.8753*	

* *p* < 0.05 (statisticaly significant).

**Table 5:** The recorded scores of PAI index during each appointment.

Group		1st app.	2nd app.			3rd app.			4th app.			5th app.		
		PAI	PAI			PAI			PAI			PAI		
		1	1	2	3	1	2	3	1	2	3	1	2	3
IA + IB	28	28	11	16	1	8	15	5	9	15	4	14	13	1
		100%	39%	57.4%	3.6%	28%	54%	18%	32%	54%	14%	50%	46.4%	3.6%
II	16	16	10	5	1	5	9	2	4	10	2	9	7	0
		100%	62%	31.7%	6.3%	31%	56%	13%	25%	62%	13%	56%	44%	0%

**Table 6:** Variation of PAI index during the whole course of orthodontic treatment with FOAs. PAI score 2 and 3 fractions (%).

Changes in PDL Space: PAI ≥ 2		
Appointment	IA + IB (*n* = 28)	II (*n* = 16)
1st	0%	0%
2nd	61%	37%
3rd	71%	69%
4th	68%	75%
5th	50%	44%
Q Cochrane (*p*) test	<0.0001	<0.0001

**Table 7:** Number of teeth with radiologically detected external apical root resorption in studied groups.

Group	Number of Teeth	1st app.	2nd app.	3rd app.	4th app.	5th app.
		Number of Teeth with EARR				
IA + IB	28	0	3	6	6	6
II	16	0	1	4	4	4

Resorption was observed in 10 out of 44 endodontically treated teeth.

**Table 8:** Characteristics of teeth with detected root resorption.

Group	Number of Teeth with EARR	Characteristics					Last Appointment (5th) Status of the Root
		Duration of Treatment (Months)		Shape of the Root	Additional Appliances	Tooth	EARR Grade
IA + IB	6	36	*T_śr_ = 21.4*	*n*	yes	11	3
		30		*n*	yes	22	2
		26		F	yes	11	2
		20		C	yes	12	2
		20		C	yes	22	3
		19		*n*	yes	21	2
II	5	18		*n*	no	35	1
		30		*n*	yes	21	2
		24		F	yes	11	1
		26		F	no	11	2

Morphological feature of the root apex: *n*-normal, F-flat, C-curved.

**Table 9:** The distribution of external apical root resorption severity evaluated during each orthodontic appointment (*n*—number of teeth).

Appointment	EARR Scale	Group		Test Ch
		IA + IB (*n* = 28)	II (*n* = 16)	
2nd	0	89.5%	94%	0.7305
	1	3.5%	0%	
	2	3.5%	6%	
	3	3.5%	0%	
3rd	0	79 %	75%	0.3956
	1	3.5%	19%	
	2	14%	6%	
	3	3.5%	0%	
4th	0	79%	75%	0.6358
	1	0%	12.5%	
	2	14%	12.5%	
	3	7%	0%	
5th	0	79%	75%	0.6358
	1	0%	12.5%	
	2	14%	12.5%	
	3	7%	0%	

**Table 10:** The percentage distribution of endodontically treated teeth with observed external apical root resorption. The additional forces used.

Appointment	EARR Score	Additional Orthodontic Forces		Test Ch Yatesa
		YES (*n* = 20)	NO (*n* = 24)	
2nd	0	80%	100%	0.0765
	>1	20%	0%	
3rd	0	60%	92%	0.0328
	>1	40%	8%	
4th	0	60%	92%	0.0328
	>1	40%	8%	
5th	0	60%	92%	0.0328
	>1	40%	8%	

**Table 11:** Changes in periodontal ligament space width in endodontically treated teeth.

PDL Space width-PAI Index					
Appointment	Additional Forces	PAI = 1	PAI = 2	PAI = 3	*p*
2nd	NO	62.5%	37.5%	0%	0.0473
	YES	30%	60%	10%	
3rd	NO	42%	58%	0%	0.0068
	YES	15%	50%	35%	
4th	NO	42%	54%	4%	0.0632
	YES	15%	60%	25%	
5th	NO	58%	42%	0%	0.5460
	YES	45%	50%	5%	

## Data Availability

Data available on request.

## Supplementary Materials

The following are available online at https://www.mdpi.com/article/10.3390/jcm10102078/s1, Table S1, STROBE Statement.
